# The outcome of isolated calf muscle vein thrombosis after open reduction and internal fixation for closed intra‐articular distal femur fractures: a prospective cohort study

**DOI:** 10.1186/s12891-021-04289-3

**Published:** 2021-04-28

**Authors:** Chen Chen, Ying Liu, Hengfu Wu, Jingmin Feng, Ao Li, Zhaozhong Wu

**Affiliations:** 1 Department of Nuclear Medicine, the People’s Hospital of Yuxi City, the Sixth Affiliated Hospital of Kunming Medical University, 653100 Yuxi, Yunnan People’s Republic of China; 2 Department of Nuclear Medicine, the Second Affiliated Hospital of Guangzhou Medicine University, No. 250 Changgang East Road, Guangdong 510260 Guangzhou, People’s Republic of China

**Keywords:** Risk factors, Calf muscle vein thrombosis, Distal femoral fracture ultrasonography, Thrombolytic therapy

## Abstract

**Background:**

To observe the outcome of isolated calf muscle vein thrombosis (ICMVT) undergoing open reduction and internal fixation (ORIF) for closed intra-articular distal femur fractures (DFFs) and to analyze related factors.

**Methods:**

The study was designed as a prospective clinical cohort study at our hospital. From August 2018 to August 2020,a total of 140 patients with flesh ICMVT after ORIF for closed intra-articular DFFs were collected during hospitalization. After the administration of antithrombotic agents immediately after diagnosis, the location and prognosis of postoperative ICMVT were examined by Duplex ultrasonography (DUS) with a three-month follow-up. There were 29 males and 111 females with the average age of 70.16 ± 8.75 years old. Sonography was used to evaluate the resolution of muscular vein thrombosis at the time point of the third month postoperatively and the results were compared between the two time points. Multivariable analysis was performed to evaluate the relationship between the resolution of ICMVT three months postoperatively and risk factors including age, Body Mass Index (BMI), gender, thrombosis length (> 5 / ≤5 cm), thrombosis diameter(> 0.6/≤0.6 cm), and thrombosis-related biochemistry indices.

**Results:**

The postoperative ICMVTs was diagnosed at 5.47 ± 2.46 days after ORIF for closed intra-articular DFFs. At the follow up of 3 months,120 cases was tending to disappear with 88 cases(62.9 %) completely dissolved and 32 cases(22.9 %) partly dissolved. There existed 14 cases (10.0 %) without change on the size and 6 cases (4.2 %) with proximal propagation. Multivariate analysis revealed that thrombus diameter over 0.6 cm (odds ratio [OR], 8.900; 95 % confidence interval [CI]: 3.623–21.865), thrombus length over 5.0 cm (OR, 3.904; 95 % CI, 1.121–13.603), FIB over 3.0 g/L (OR, 3.627; 95 % CI, 1.356–9.689), and D-dimer over 1.0 mg/L (OR, 2.602; 95 % CI, 1.075–6.296) were four independent risk factors of non-completely dissolved ICMVTs.

**Conclusions:**

85.8 % of ICMVT was tending to disappear at the third months after ORIF for closed intra-articular DFFs. Thrombus diameter, thrombus length, FIB, and D-dimer were four independent risk factors of non-completely dissolved ICMVTs. The Thrombus diameter has a significant effect on the natural course of ICMVTs, especially with diameter larger than 0.6 cm.

## Background

Intermuscular veins of lower limb are one of the most common thrombosis sites after major orthopedic surgery [1], which involve soleus veins, gastrocnemius veins and communicating branches between them. The soleus veins usually drain into the posterior tibial veins and peroneal veins, whereas the gastrocnemius veins drain into popliteal veins [2, 3]. Intermuscular vein thrombosis is located in the distal (calf) veins, which can readily be neglected in clinics due to its lack of specific manifestations. The role of isolated calf muscle vein thrombosis (ICMVT) remains controversial [4]. Most of the ICMVT gradually disappear, but there remains a proportion of the ICMVT progressing rapidly to deep venous thrombosis (DVT) or pulmonary embolism (PE) [5]. Whether or not the timing, dose, and duration of the thrombolytic therapy for ICMVT should be referred to those for thrombosis occurring at other sites is also debatable [5]. A randomized controlled study did not show superiority of a short-term regimen of low-molecular-weight heparin and compression therapy in comparison with compression therapy alone in patients with ICMVT [4]. A vascular laboratory studies of 406 patients with ICMVT indicated that isolated gastrocnemius and soleus vein thrombosis (VTE) was associated with a clinically significant rate of venous thromboembolism which was dramatically reduced with therapeutic anticoagulation [6]. In an outpatient clinic, a prospective study on consecutive 128 patients with ICMVT demonstrated that pulmonary embolism (PE) at the ICMVT initial diagnosis was not rare, and mid-term follow-up (mean, 26.7 months) revealed that 18.8 % of patients had at least one VTE recurrence. The treatment of acute ICMVT needed to be standardized because no guidelines currently existed [7].

There are many factors leading to the spread of ICMVT. Tumor is considered to be an important factor in the progression of ICMVT to the proximal end [8]. Regarding patients with end stage renal disease or stroke, the ICMVT was easier to spread [5]. Further, some scholars thought that though, as reported, PE during surveillance was the most precisely way to reflect PE in ICMVT, but such a risk of PE should not just be ignored and its importance still needs to be discussed [9]. Well-designed and adequately powered studies are still needed to give strong evidence to recognize the real risk of ICMVT to distinguish the one with high risk and determine appropriate way to treat it. After investigating 120 legs of 60 autopsy cases with fatal pulmonary thromboembolism, Norimasa et al. found that the soleal vein was the most frequent site of deep vein thrombosis, and anatomical characteristics and physiological mechanisms played a major role in the occurrence and propagation of venous thrombi [3]. Another study of 58 lower limbs in 29 patients showed that ICMVT was a pulmonary embolic source when isolated thrombosis of the large soleal vein was more than 7 mm in diameter [10]. The studies above have important clinical implications for evaluating the progression of ICMVT, but few focused on the patients diagnosed with closed intra-articular distal femur fractures (DFF) undergoing open reduction internal fixation (ORIF).

Distal femur fractures (DFFs) comprise of approximately 8.7 and 0.8 % of all femoral fractures and body fractures in Chinese adults, respectively [11]. Besides, DFFs frequently involve articular surface and may be associated with vascular or nerve injuries. Given the above potential involvements, DFFs were prone to being complicated with traumatic arthritis, venous thromboembolism (VTE), or other perioperative complications. Notwithstanding, deep venous thrombosis (DVT) poses a significant challenge for most orthopedic and trauma surgeons as the immune, and inflammatory systems are activated, keeping the intravascular system in a hypercoagulability state, thus, leaving the patient at a higher risk of developing DVT. The one-month mortality of DVT was 4.6 %, much higher as compared to that of the general population [12]. Several studies have focused on issues concerning incidence, predilection sites, and related predictors of DVT after hip fractures [13, 14], joint arthroplasty [15, 16], and ankle trauma [17, 18], however, the studies above are nonetheless limited in areas such as insufficient ICMVT data and the short track of postoperative prognosis. Importantly, the literature on the epidemiological characteristics of ICMVTs after ORIF for closed intra-articular DFFs is rare, especially over the past decade. More so, the prophylactic treatment for ICMVTs remains controversial, and a portion of ICMVTs cases typically finally propagated proximally [19].

Thus, we decided to carry out a prospective study which was designed mainly for two primary purposes: first, to analyze the distribution of the clinical outcome of the ICMVTs after ORIF for closed intra-articular DFFs with a three-month follow-up; and secondly, to evaluate the relationship between the resolution of ICMVTs 3 months postoperatively and potential risk factors.

## Materials and methods

### Study design

**Fig. 1:**
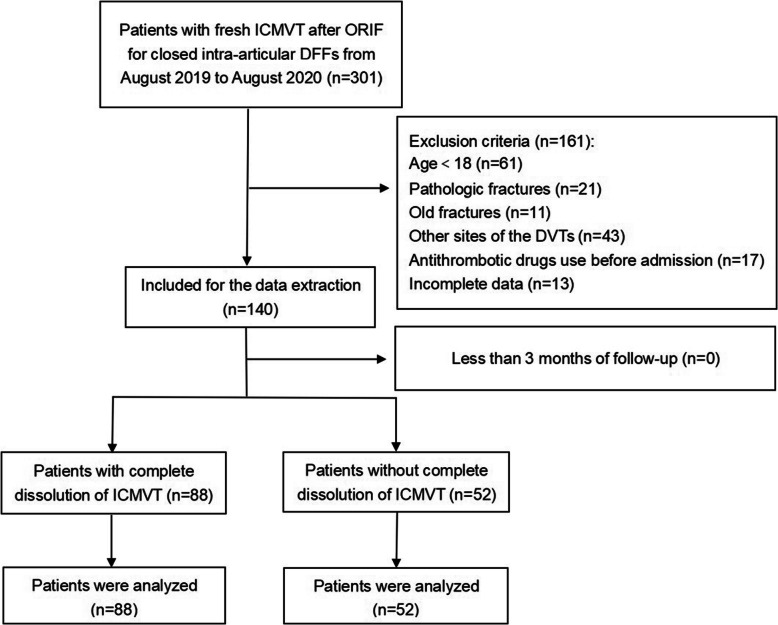
The flow chart for the selection of study participants

The present study, which was conducted at our hospital, is a prospective single-center study involving a total of 140 patients with flesh ICMVT after ORIF for closed intra-articular DFFs. The study protocol was carried out according to the Declaration of Helsinki and approved by the Institutional Review Board (NO 2020kmykdx6h20). All participants in the study had signed a written informed consent before the study was conducted. The exclusion criteria are listed as follows: (i) < 18 years of age; (ii) with other sites of the DVTs at initial diagnosis; (iii) open or pathological fractures; (iv) previous thromboembolism history; and (V) antithrombotic drugs use before admission (low molecular weight heparin and others), and patients with incomplete medical records. As presented in Figure. 1, a total of 140 participants with closed DFFs were finally enrolled.

### Definition and detection of ICMVT

Duplex ultrasonography (DUS) was performed to diagnose ICMVT according to the Robinov group’s criteria [20]. The criteria for the diagnosis of ICMVT were: non compressed vein, lumen obstruction or filling defect, the lack of respiratory vibration above the knee vein segment, and inadequate flow augmentation to the calf. DUS was used to scan participants for bilateral lower-extremity ICMVTs once they were admitted. Administration of low-molecular weight heparin (LMWH) within 24 h of presentation. The LMWH administered was enoxaparin (40 mg daily therapeutic or prophylactic thromboembolic agents are routinely given. After that, participants would be retested by DUS every three days. The outcome of the ICMVTs were classified into two groups: completely dissolved (CD) group, and non-completely dissolved (non-CD) group. The non-CD group involved three types: part dissolution, no change, and proximal propagation.

### Data acquisition and variables of interest

Three orthopedic surgeons with unified training recorded the data mentioned below. The surgeons closely observed the patients during morning ward rounds while, however, reviewing the patient’s clinical data. The outcomes of ICMVT was followed within three months after admission. Complex variables of interest were categorized to three aspects.

Demographic variables involved age (years), gender, body mass index (BMI, kg/m^2^), cigarette consumption, alcohol consumption, diabetes mellitus, hypertension, cardiovascular disease, and previous surgeries at any part of the body.

Intra-articular fracture-related variables included fracture type according to AO/OTA classification system, concurrent fracture sites (single fracture and multiple fractures), the American Society of Anesthesiologists (ASA, I-II, and III-IV) score, and injury mechanisms. The injury mechanisms were grouped to two categories: low-energy (fall from a standing height) and high-energy (automobile accidents, falling from height, and others).

Thrombosis-related variables were collected as follow: thrombus length, maximum diameter of thrombus. Thrombosis-related biochemistry indices on the day of first diagnosed as ICMVT involved hemoglobin concentration (HGB,g/L), red blood cell count (RBC, 10^9^/L), white blood cell count (WBC, 10^9^/L), blood platelet count (PLT, 10^9^/L), prothrombin time (PT, s), activated partial thromboplastin time (APTT, s), fibrinogen (FIB, g/L), and D-dimer (mg/L).

### Statistical analysis

All statistical analyses were performed in SPSS version 25.0 (IBM Corp., Armonk, NY, USA). Continuous factors were presented as means ± standard deviations (SD). We assessed normality of our data using the Shapiro-Wilk test, then performed Whitney U or t tests, to compare continuous factors between DVT groups and controls. Continuous factors with statistical significance were subjected to receiver operating characteristic (ROC) analyses to obtain optimum cut-off values, which was calculated by maximizing the sum of sensitivity and specificity in the ROC curve. These cut-off values were then used to convert continuous variables into categorical variables prior to logistic regression. The relationships between each categorical variable and the rate of complete dissolution of ICMVT three months postoperatively were analyzed using a Pearson chi-square test. Factors with statistical significance, from univariate analysis, were subjected to multiple logistic regression analyses (backward LR). Models were checked using Hosmer-Lemeshow’s goodness of fit tests. Data followed by *p* value < 0.05 were considered statistically significant.

## Results

### Participants selection

Figure 1 shows the flow chart for the screening of the study participants. During the investigation, a total of 301 patients were performed ORIF for closed intra-articular DFFs in our institution. Amongst the postoperative patients, a total of 61 patients were < 18 years of age; 21 had pathological fractures (including bone or joint tumor, or soft tissue tumor); 11 had old fractures;43 had the DVTs in other sites; 17 used antithrombotic drugs before admission, while 13 patients had an incomplete clinical data. A total of 140 patients were finally enrolled in the study without lost to follow-up. The average age of the enrolled patients was 70.16 ± 8.75 years, while the average BMI was 25.91 ± 4.36 kg/m^2^.

### Features of the postoperative ICMVTs

**Fig. 2:**
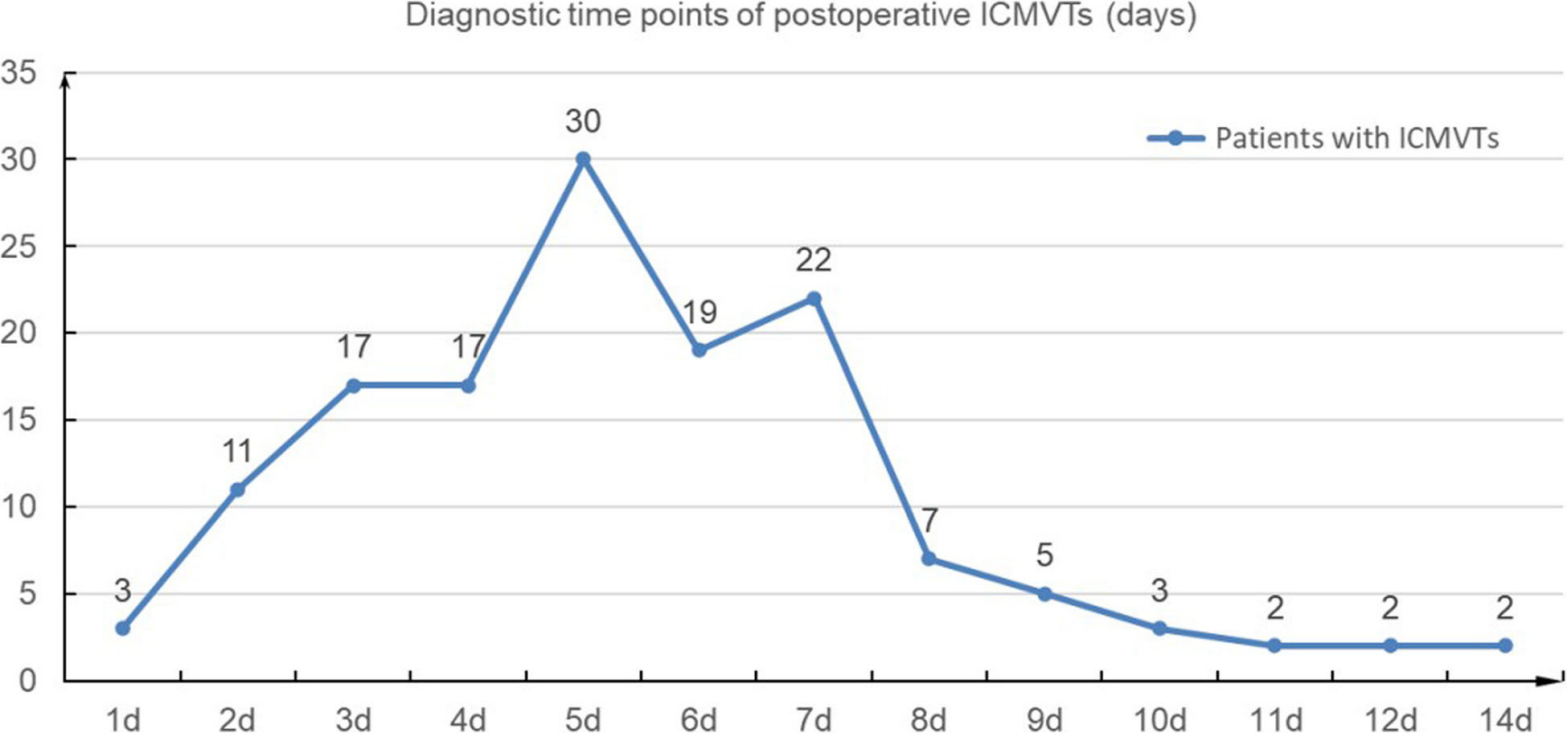
Diagnostic time points of postoperative isolated calf muscle vein thrombosis (ICMVTs) after injuries (n = 140)

Figure 2 shows the diagnostic time points of postoperative ICMVTs. The postoperative ICMVTs was diagnosed at 5.47 ± 2.46 days after ORIF. After the administration of low-molecular weight heparin (LMWH), 62.9 % (*n* = 88) of the ICMVTs was completely recanalized.

### Continuous factors and the optimum cut‐off value

Table 1 shows the comparison of continuous variables between completely dissolved (CD) group and non-completely dissolved (non-CD) group. Summarily, we observed significant differences with regards to thrombus length, thrombus diameter, FIB, and D-dimer. In addition, ROC values across the four continuous variables revealed statistical significances (Table 2). The optimum cut-off values for the thrombus length, thrombus diameter, FIB, and D-dimer were 5 cm, 0.6 cm, 3.0 g/L, and 1.0 mg/L, respectively. We used these cut-off values to divide the four factors into two categories.

**Table 1:** Comparison of continuous variables between completely dissolved (CD) group and non-completely dissolved (non-CD) group

Variables	CD group (mean ± SD) (*n* = 88)	Non-CD group (mean ± SD) (*n* = 52)	*P*-value
Age (years)	70.32 ± 8.88	69.88 ± 8.60	0.761^b^
BMI (kg/m^2^)	26.16 ± 4.67	25.48 ± 3.80	0.427^b^
Preoperative hospital stay (days)	6.79 ± 4.43	8.31 ± 4.77	0.110^b^
Operation duration (minutes)	152.10 ± 55.47	164.62 ± 69.60	0.221^b^
Intraoperative blood loss (mL)	504.66 ± 332.92	507.33 ± 453.14	0.662^b^
Thrombus length (cm)	3.75 ± 0.75	4.05 ± 1.04	0.034^b^*
Thrombus diameter (cm)	0.48 ± 0.14	0.68 ± 0.19	0.000^b^*
HGB (g/L)	110.80 ± 12.41	109.75 ± 13.37	0.637^a^
RBC (10^9^/L)	3.32 ± 0.55	3.46 ± 0.50	0.202^b^
WBC (10^9^/L)	8.77 ± 3.14	9.17 ± 3.02	0.462^a^
PLT (10^9^/L)	249.87 ± 101.06	244.93 ± 80.66	0.985^b^
TP (g/L)	58.48 ± 7.25	59.20 ± 6.56	0.361^b^
ALB (g/L)	32.66 ± 5.15	33.82 ± 4.84	0.057^b^
PT (s)	11.95 ± 1.37	11.82 ± 0.84	0.904^b^
APTT (s)	29.32 ± 4.23	30.39 ± 4.62	0.130^b^
FIB (g/L)	3.40 ± 0.96	4.00 ± 1.13	0.001^a^*
D-dimer (mg/L)	2.13 ± 3.80	2.43 ± 2.86	0.015^b^*

*Statistical significance; ^a^ Student t test; ^b^ Mann-Whitney U test

*ICMVTs* isolated calf muscle vein thrombosis; *BMI* body mass index; *HGB* hemoglobin; *RBC* red blood cell; *WBC* white blood cell; *PLT* platelet; *TP* total protein; *ALB* albumin; *PT* prothrombin time; *APTT* activated partial thromboplastin time; *FIB* fibrinogen

**Table 2:** The ROC curve analysis of continuous variables with statistical significance

Variable	Cut-off value	Area under the curve (95 % CI)	Sensitivity	Specificity	*P*-value
Thrombus length (cm)	5.0	0.607(0.506–0.708)	26.9 %	93.2 %	0.035
Thrombus diameter (cm)	0.6	0.819(0.743–0.894)	80.8 %	71.6 %	0.000
FIB (g/L)	3.0	0.651(0.556–0.747)	76.9 %	48.9 %	0.003
D-dimer(mg/L)	1.0	0.623(0.530–0.717)	69.2 %	55.7 %	0.015

*ROC* receiver operating characteristic; *CI* confidence interval; *FIB fibrinogen*

### Univariate analysis of the categorical factors

Univariate analysis of categorical factors revealed significant differences among the 20 factors across the two groups with regards to thrombus length (> 5.0 cm), thrombus diameter (> 0.6 cm), ALB (< 35 g/L), FIB (> 3.0 g/L), and D-dimer (> 1.0 mg/L) (Table 3). Consequently, we used the 5 factors into the multivariate regression analysis.

**Table 3:** Comparison of categorical variables between completely dissolved (CD) group and non-completely dissolved (non-CD) group

Variables	Number (%) of patients in CD group (*n* = 88)	Number (%) of patients in non-CD group (*n* = 52)	*P*-value
Gender (males)	14(16.0)	15(28.8)	0.068
Living place (rural)	58(65.9)	35(67.3)	0.866
Diabetes mellitus	31(35.2)	17(32.7)	0.760
Hypertension	38(43.2)	21(40.4)	0.746
Cardiovascular diseases	26(29.9)	17(32.7)	0.729
Previous surgery in any site	29(33.3)	15(28.8)	0.582
Smoking	4(4.5)	5(9.6)	0.237
Alcohol consumption	5(5.7)	7(13.5)	0.112
Injury mechanisms (high energy)	41(46.6)	25(48.1)	0.865
Fracture side (left)	47(53.4)	30(57.7)	0.623
Fracture classification			0.114
Type B	56(63.6)	26(50.0)	
Type C	32(36.4)	26(50.0)	
ASA score			0.276
I-II	49(55.7)	24(46.2)	
III-V	39(44.3)	28(53.8)	
TP(< 60 g/L)	50(56.8)	22(42.3)	0.097
Thrombus length (> 5.0 cm)	6(6.8)	14(26.9)	0.001*
Thrombus diameter (> 0.6 cm)	14(15.9)	34(65.4)	0.000*
ALB(< 35 g/L)	65(73.9)	30(57.7)	0.048*
PT(> 12.5 s)	19(21.6)	7(13.5)	0.232
APTT(< 28 s)	32(36.4)	16(30.8)	0.500
FIB(> 3.0 g/L)	48(54.5)	41(78.8)	0.004*
D-dimer(> 1.0 mg/L)	38(43.2)	33(63.5)	0.020*

*Statistical significance

*ICMVTs* isolated calf muscle vein thrombosis; *ASA* the American Society of Anesthesiologists; *TP* total protein; *ALB* albumin; *PT* prothrombin time; *APTT* activated partial thromboplastin time; *FIB* fibrinogen

### Multiple logistic regression analysis

The adjusted data from multivariate regression analysis is summarized in Table 4. Briefly, thrombus diameter over 0.6 cm (odds ratio [OR], 8.900; 95 % confidence interval [CI]: 3.623–21.865), thrombus length over 5.0 cm (OR, 3.904; 95 % CI, 1.121–13.603), FIB over 3.0 g/L (OR, 3.627; 95 % CI, 1.356–9.689), and D-dimer over 1.0 mg/L (OR, 2.602; 95 % CI, 1.075–6.296) were all independent risk factors of non-completely dissolved ICMVTs. The Hosmer-Lemeshow test further revealed excellent goodness of fit (χ^2^ = 3.986; *p* = 0.781).

**Table 4:** Multivariate analysis of factors associated with non-completely dissolved ICMVTs

Variables	Odds ratio	95 % CI	*P*-value
Thrombus diameter (> 0.6 cm)	8.900	3.623–21.865	0.000
Thrombus length (> 5.0 cm)	3.904	1.121–13.603	0.032
FIB(> 3.0 g/L)	3.627	1.356–9.689	0.010
D-dimer(> 1.0 mg/L)	2.602	1.075–6.296	0.034

*ICMVTs *isolated calf muscle vein thrombosis*; CI* confidence interval; *FIB* fibrinogen

## Discussion

Previous literature stated that there were two main pathways of proximal progression of isolated calf muscle vein thrombosis. On the one hand, ICMVT can spread to the peroneal vein and posterior tibial vein. On the other hand, the ICMVT can spread directly to the popliteal vein[2]. In the present study, we observed the outcome of ICMVT after ORIF for closed intra-articular DFFs. Our results showed that 85.8 % ICMVT was tending to disappear at the third months after operation. Longer follow-up results in the literature suggested thromboprophylaxis after knee surgery could completely disappear over time. Kim et al. studied 227 patients after total knee arthroplasty who underwent primary total knee arthroplasty for the incidence and natural history of deep-vein thrombosis. He found that further venograms at six months after operation in all 143 limbs which had thrombi showed that all had completely resolved regardless of the size or location[21]. The uniform follow-up of our study was 3 months after ORIF, and then only six cases were found to be complicated with popliteal vein thrombosis. The rate of proximal propagation was 4.2 % (6/140), which was related to the shorter duration of the follow-up period.

After adjusting for confounding variables, thrombus length, thrombus diameter, FIB, and D-dimer were shown to be independent risk factors of non-completely dissolved ICMVTs. Thrombus size was a factor not negligible in the process of thrombus dissolution. Previous data indicated that the diameter of intermuscular vein had a significant effect on thrombosis[4–6]. Chen et al. retrospectively reviewed 1461 patients who underwent varicose vein surgery with a tourniquet, and gastrocnemius vein dilation (gastrocnemius veins diameter over 0.5 cm) had the highest predictive power for postoperative DVT [22]. Yamaki et al. studied 68 patients undergoing total knee or total hip arthroplasty, and he found a gastrocnemius vein diameter > 0.25 cm seemed to contribute to the development of postoperative DVT [23]. From the information above, we can find that at present, there remains no uniform critical value for the diameter of blood vessels and the size of thrombus. In our present study, with the ROC analysis, we calculated that both thrombus length over 5.0 cm and thrombus diameter over 0.6 cm were the significance cutoff value. Regarding to patients with ICMVTs diameter over 0.6 cm, it may be helpful to take appropriate Anticoagulant treatment for them even after discharge.

D-dimer value is a well-known risk factor for thrombosis as extensively studied by several pieces of orthopedic literature [24, 25]. Notably, we found out that an optimum cut-off value of D-dimer value was 1.0 mg/L, which was about twice that of the standard upper limit value (0.5 mg/L). Furthermore, the D-dimer value over 1.0 mg/L was related to a 2.60 times increased risk of non-completely dissolved ICMVTs. What should be taken into consideration is that isolated D-dimer value tends to have high sensitivity, but often, the specificity is low in prediction of DVTs. In a derivation study of the age-dependent cut-off value used in the current analysis, it was shown that the optimal D-dimer cut-off value gradually increases as age increases [26]. Compared with D-dimer, the FIB volume is a more common laboratory marker strongly correlated with coagulation function. With the present study, we found out that patients with FIB over 3.0 g/L had a 3.63-fold risk of non-completely dissolved ICMVTs. However, Faunø et al. found the sensitivity of fibrinogen scanning to be 44 % for the non-operated limb and 50 % for the calves. They also concluded that the use of fibrinogen uptake test as single diagnosticum is not valid and can only be recommended in combination with phlebography [27].

The present study had three key highlights: firstly, it was the largest prospective cohort of closed intra-articular DFFs patients diagnosed by DUS for ICMVTs. Secondly, feature analysis and prognosis of ICMVTs over three months were performed. Thirdly, ROC analysis was conducted to identify a highly sensitive cut-off value for continuous variables, and both thrombus diameter over 0.6 cm and thrombus length over 5.0 cm were uncommon independent protective factors for non-completely dissolved ICMVTs. Nonetheless, the study had some limitations. Firstly, the present research was a single-center study, which might not, however, represent prevalent populations. Secondly, the follow-up time was relatively short, and a longer follow up might be needed. Finally, some variables that potentially influence the dissolution of ICMVTs were not included, such as hidden blood loss or the tumor history.

## Conclusions

85.8 % of ICMVT was tending to disappear at the third months after ORIF for closed intra-articular DFFs. Thrombus diameter, thrombus length, FIB, and D-dimer were four independent risk factors of non-completely dissolved ICMVTs. The Thrombus diameter has a significant effect on the natural course of ICMVTs. ICMVTs with diameter larger than 0.6 cm are difficult to be resolved thoroughly.

## Data Availability

The datasets used and analysed during the current study are available from the corresponding author upon reasonable request.

## Abbreviations

ICMVTs: Isolated calf muscle vein thrombosis
BMI: Body mass index
HGB: Hemoglobin
RBC: Red blood cell
WBC: White blood cell
PLT: Platelet
TP: Total protein
ALB: Albumin
PT: Prothrombin time
APTT: Activated partial thromboplastin time
FIB: Fibrinogen
ROC: Receiver operating characteristic
CI: Confidence interval
ASA: The American Society of Anesthesiologists
LMWH: Low-molecular weight heparin
